# Expression of Matrix Metalloproteinase-7 Predicts Poor Prognosis in Gastric Cancer

**DOI:** 10.1155/2022/2300979

**Published:** 2022-04-22

**Authors:** Wareeporn Wattanawongdon, Theeraya Simawaranon Bartpho, Taweesak Tongtawee

**Affiliations:** Suranaree University of Technology, Nakhon Ratchasima, Thailand

## Abstract

To date, no potential markers have been established for predicting prognosis in gastric cancer. Matrix metalloproteinase-7 (MMP-7) has been suggested as a prognostic marker in several cancers. In this study, we aimed to determine the expression of the MMP-7 protein and its polymorphisms in gastric cancer tissues. The association between MMP-7 expression level and clinicopathological characteristics was also evaluated. MMP-7 protein expression and its polymorphisms were investigated in a total of 400 patients using immunohistochemistry and TaqMan SNP genotyping assays. The correlation of MMP-7 expression with clinicopathological characteristics, including tumor location, tumor size, histologic type, lymphatic invasion, vascular invasion, pathological T stage, pathological TNM stage, residual tumor, and CEA level, was investigated. Odds ratios (ORs) and 95% confidence intervals (CIs) were calculated using a multivariate Cox proportional hazards regression model. MMP-7 expression was found in 283 of 400 (70.75%) gastric cancer tissues. Expression of MMP-7 was significantly associated with poor clinicopathological characteristics, including vascular invasion (OR = 6.61, 95%CI = 4.26–9.89, *p* = 0.024), lymphatic invasion (OR = 8.17, 95%CI = 4.47–12.39, *p* = 0.017), undifferentiated histologic type (OR = 2.46; 95% CI, 1.31–4.52; *p* = 0.014), higher TNM stage (stage IV) (OR = 1.48, 95%CI = 1.08–3.08, *p* = 0.047), and high CEA level (OR = 5.96, 95%CI = 2.12–8.12, *p* = 0.026). We further observed a significant association of the variant genotype; gastric cancer patients carrying GG of MMP-7 (-181A/G; rs11568818) had a greater increased risk of MMP-7 expression than did wild-type (WT) carriers (AG: odds ratio (OR) = 5.67; 95%CI = 1.57–7.23; *p* = 0.024 and GG: OR = 8.32; 95%CI = 2.94–11.42; *p* = 0.016). These findings suggest that MMP-7 expression can be used to predict the prognosis of gastric cancer patients.

## 1. Introduction

Gastric cancer is still a major health problem and a major cause of cancer mortality worldwide. The incidence of gastric cancer has constantly increased over the last decades, particularly in East Asia [1]. Although the mortality rate associated with gastric cancer has improved over time, the 5-year survival is still less than 30% [2]. Most gastric cancer patients are diagnosed at advanced stages for which no potential treatments are available. Surgical resection at an early stage is currently the only effective treatment option in clinical practice. Patients with cancers at the same stage often differ in their survival time and in their response to therapeutic approaches [3]. In most cases, differences in already known clinicopathological and prognostic outcomes appear to explain such heterogeneity [4]. The availability of biomarkers that can be used to predict poor prognosis in patients appears to be one of the most promising approaches for improving cancer survival rates. Although several candidate biomarkers for gastric cancer have been reported, none of these can be used in clinical practice. Thus, new biomarkers that better predict prognosis in gastric cancer patients should be identified.

Tumor cells invade the basement membrane by secreting enzymes that degrade the extracellular matrix (ECM) and cause cellular adhesions. These enzymes are called matrix metalloproteinases (MMPs). MMPs belong to a family of Zn^2+^-dependent endopeptidases that are closely associated with tumorigenesis, invasion, and metastasis [5]. MMPs can be divided into five divergent groups based on their substrate specificities and domain structures; these groups include collagenases, matrilysins, stromelysins, gelatinases, and membrane-type MMPs [6]. Matrix metalloproteinase-7 (MMP-7), also known as matrilysin, is the smallest member of the MMP family. Numerous studies have demonstrated that MMP-7 plays an important role in multiple processes associated with tumor progression, including cell growth, invasion, metastasis, and angiogenesis. MMP-7 is capable of degrading a broad range of substrates in the ECM, including type IV collagen, fibronectin, proteoglycans, vitronectin, laminin, gelatin, elastin, and entactin [7]. Overexpression of MMP-7 has been found in several human cancers, including breast [8], colon [9], esophageal [10], and gastric cancer [11]. In addition, MMP-7 expression has been shown to be associated with an aggressive malignant phenotype and worse prognosis in patients with gastric cancer [12].

Accumulated evidence indicates that variation in the DNA sequence of the MMP-7 gene may result in variable expression of MMP-7 in different individuals. The variant 181A ⟶ G (rs11568818), which is found in the MMP-7 promoter region, is known to modulate the expression of genes and proteins and has been studied in various disease conditions, including cancers [13]. Although MMP-7 has been suggested as a candidate marker in gastric cancer, the reports are inconsistent, with some studies showing that MMP-7 expression does not correlate with the survival of patients with gastric cancer. Hence, we aimed to determine whether MMP-7 expression can be used as a prognostic biomarker in gastric cancer patients. To do this, we measured the expression of MMP-7 protein and its polymorphisms (rs11568818) in gastric cancer tissues. The association between MMP-7 expression level and clinicopathological characteristics was also evaluated to assess the value of MMP-7 as a possible candidate marker for predicting individuals with higher risk of developing gastric cancer.

## 2. Materials and Methods

### 2.1. Patients and Tissue Samples

Four hundred gastric cancer patients, who underwent surgical resection at Suranaree University of Technology Hospital, Buriram Hospital, and Surin Hospital between January 2012 and December 2018, were included in this study. Written informed consent was obtained from all patients prior to study entry. The study was approved by the Ethics Committee for Research Involving Human Subjects, Suranaree University of Technology (EC-62-0003). The study was conducted in accordance with the ethical guidelines set forth in the Declaration of Helsinki. All tissues were fixed in 10% formalin, embedded in paraffin wax, and cut into 4-*μ*m sections. Each specimen block was stained with hematoxylin and eosin (H&E) for histological examination, and representative blocks were chosen for immunohistochemical study. Additionally, all patients were clinically evaluated according to TNM staging for gastric cancer developed by the American Joint Committee on Cancer (seventh edition, 2010) (14) for tumor stage, histologic grade, and metastasis.

### 2.2. DNA Extraction

Genomic DNA was extracted from paraffin-embedded (FFPE) tissue of 400 gastric cancer patients using a QIAamp DNA FFPE Tissue Kit (Qiagen, Duesseldorf, Germany) according to the manufacturer's instructions. Briefly, the paraffin-embedded tissues were deparaffinized in xylene, hydrated in 100% ethanol, and digested with lysis buffer and proteinase K. Genomic DNA was purified from the tissue lysate using QIAamp spin columns. The concentration and purity of the isolated DNA and were evaluated using a DS-11+ spectrophotometer (Denovix, Wilmington, DE, USA) and stored at −20°C.

### 2.3. MMP-7 Polymorphism

To characterize MMP-7 gene polymorphism (-181A/G; rs11568818), a custom TaqMan SNP Genotyping Assay was conducted. Polymorphisms of MMP-7were selected according to the SNP database of the National Center for Biotechnology Information. The MMP-7 genotype was determined by TaqMan allelic discrimination using a predesigned Custom TaqMan SNP Genotyping Assay and real-time PCR. Forward and reverse primers were used along with the wild-type probe VIC and probes FAM used for the variant allele. The primers and probes were supplied by Applied Biosystems. Real-time PCR was performed using a LightCycler 480 II instrument (Roche Diagnostics, Neuilly sur Seine, France) according to the manufacturer's instructions. Amplification was performed using an initial cycle of 60°C for 1 min and 95°C for 10 min followed by 40 cycles of 95°C for 15 s and 60°C for 1 min. The success rate of genotyping was greater than 94%. Negative controls and duplicate samples were used to check the accuracy of genotyping. The PCR results were initially analyzed using LightCycler 480 Software 1.5 (Roche Diagnostics, Neuilly sur Seine, France).

### 2.4. Immunohistochemistry

Immunohistochemical evaluation of MMP-7 expression was performed on 4-*μ*m-thick gastric cancer tissue sections using the avidin-biotin complex method (ABC; Thermo Fisher, IL, USA). All sections were deparaffinized through a xylene series and then rehydrated by passage through a series of graded alcohol solutions (100, 95, 80, and 70%) in water. The sections were unmasked by heating them in 10 mM citrate buffer and pH 6.0 in a microwave oven at 500 W for 5 min and were then washed with phosphate-buffered saline (PBS) before incubation in 5% normal serum for 1 h at room temperature to block nonspecific background reactions. The slides were then incubated with a mouse monoclonal antibody against MMP-7 (1 : 200, clone sc-80205; Santa Cruz Biotechnology, Inc.) overnight at 4°C in a moist chamber. Following washes with PBS, the sections were incubated with A biotinylated goat antimouse secondary antibody (1 *μ*g/ml) for 30 min, followed by incubation with ABC HRP-conjugated avidin-biotin-complex (Thermo Fisher, Rockford, IL, USA) for 10 min. The color was developed using an aminoethyl carbazole substrate solution (Life Technologies Corporation, Carlsbad, CA, USA). The sections were counterstained with Mayer's hematoxylin and evaluated under a light microscope.

### 2.5. Immunohistochemical Evaluation

The slides were independently examined by three experienced pathologists who were blinded to the clinicopathologic data of the patients. The discrepancy between the pathologists' analyses was minimal and, when present, was resolved by consensus. The immunohistochemical evaluation of MMP-7 staining was based on the percentage of positively stained cells; under high magnification (×200), 500 cells per field in at least five different fields were counted; and the samples were scored as follows: lack of staining was scored as 0; samples in which 1–10% of the cells were stained were scored as 1+; samples in which >10 and ≤ 50% of the cells were stained were scored as 2+; and samples in which >50% of the cells were stained were scored as 3+. Cases classified as 0 were defined as negative, whereas MMP-7 expression was considered positive when scores were ≥ 1.

### 2.6. Statistical Analysis

The Statistical Package for Social Sciences (SPSS®) version 20 software (IBM, Armonk, NY, USA) was used to statistically analyze the data. The statistical significance of any associations between the MMP-7 polymorphism and MMP-7 protein immunohistochemical staining and the patient's clinicopathological characteristics (sex, age, underlying conditions, family history of gastric cancer, tumor location, tumor size, lymphatic invasion, vascular invasion, histologic differentiation, pathological T stage, TNM staging, residual tumor, and carcinoembryonic antigen (CEA) level was determined using the *χ*^2^ (chi-squared) test or Fisher's exact test. To assess the prognostic index, we first analyzed the data using a univariate logistic regression model. Parameters that were significant in the univariate analysis were then assessed in the final analysis using a multivariate logistic regression model with a stepwise forward selection methodology. *p* values <0.05 were considered statistically significant.

## 3. Results

### 3.1. Patient Characteristics

The demographic data and MMP-7 statuses of the gastric cancer patients in the study are summarized in Table 1. The study included 190 males and 210 females ranging in age from 40 to 83 (median = 62.89) years. No statistically significant differences with respect to age, sex, or underlying conditions were found among the samples that showed low versus high MMP-7 expression. In the gastric cancer patients in our sample, the frequency of high MMP-7 expression was significantly higher than the frequency of low MMP-7 expression (Table 1).

### 3.2. Association between MMP-7 Polymorphism and Protein Expression in Gastric Cancer Patients

Table 2 presents the genotype patterns of the MMP-7 gene SNP and the OR estimates of gastric cancer risk for each genotype. The MMP-7 wild-type genotype (AA) was observed in 69% of the 400 gastric cancer patients in our study, whereas 17% were heterozygous (AG) and 14% were homozygous (GG) for the mutation. The AA, AG, and GG genotypes were found in 42.75%, 9.5%, and 12%, respectively, of the samples with high MMP-7 expression. We further observed a significant association between presence of the GG genotype of MMP-7 rs11568818 and high MMP-7 expression compared to the wild-type (AA) and AG carriers (OR = 5.87; 95%CI = 3.73–9.24; *p* = 0.035).

### 3.3. Association between MMP-7 Expression and Clinicopathological Characteristics

**Figure 1:**
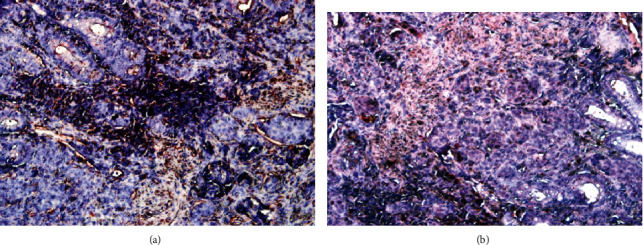
Immunohistochemical staining of gastric cancer specimens for MMP-7. (a) MMP-7 low expression. (b) MMP-7 high expression in gastric cancer. The red color indicates the presence of MMP-7 protein in gastric cancer specimens (magnification ×200).

A total of 400 gastric cancer cases were examined; of these, 176 patients had differentiated adenocarcinoma (well to moderately differentiated adenocarcinoma), and 224 patients had undifferentiated adenocarcinoma (poorly differentiated adenocarcinoma) according to TNM staging. Tumor location was analyzed; 60 patients were classified as having upper gastric cancer, 116 were classified as having middle gastric cancer, and 224 were classified as having lower gastric cancer. Using the optimal cutoff point of 70 mm, 221 cases were categorized as small gastric cancer, and 179 cases were categorized as large gastric cancer (≥70 mm). Lymphatic invasion was observed in 237 cases, and 259 cases presented vascular invasion. Pathological TNM stages I, II, III, and VI were found in 29, 57, 138, and 176 patients, respectively. Residual tumors were found in 31 cases. Of these 31 cases, 11 had microscopic tumors, and 20 had macroscopic residual tumors. High serum levels of CEA ≥ 5.0 (ng/ml) were found in 232 patients. MMP-7 expression, which appeared as red staining, was distributed heterogeneously in the cytoplasm of cancer cells (Figure 1). Positive MMP-7 expression was found in 283 of the 400 cases (70.75%) and was observed to be significantly associated with undifferentiated adenocarcinoma, lymphatic invasion, vascular invasion, higher TNM staging (stage IV), and high CEA levels (Table 3). However, no association between MMP-7 expression and other clinicopathological outcomes, including tumor location, tumor size, pathological T stage, and residual tumor, was observed (*p* > 0.05) (Table 3). The variables that were significantly associated with MMP-7 expression in the univariate analysis were further analyzed using a multivariate logistic regression model to determine the independent prognostic value of the expression of MMP-7 and to control for other prognostic factors such as age, sex, and underlying disease. The results showed that undifferentiated histologic type (OR = 2.46; 95% CI, 1.31–4.52; *p* = 0.014), lymphatic invasion (OR = 8.17; 95% CI, 4.47–12.39; *p* = 0.017), vascular invasion (OR = 6.16; 95% CI, 4.26–9.89; *p* = 0.014), higher TNM staging (stage IV) (OR = 1.48; 95% CI, 1.08–3.08; *p* = 0.047), and higher CEA level (OR = 5.96; 95% CI, 2.12–8.12; *p* = 0.026) were significantly associated with positive MMP-7 staining (Table 4).

## 4. Discussion

To date, no effective biological markers have been established for predicting the prognosis of gastric cancer in clinical practice. Several studies have demonstrated that expression of MMP-7 protein may serve as a prognostic marker in multiple types of cancers [14, 15]. Moreover, a specific polymorphism of MMP-7 (rs11568818) modulates protein expression and possibly affects cancer progression. Here, we aimed to examine whether the expression of MMP-7 and the presence of this polymorphism can be used to predict the prognosis of gastric cancer. Our results were obtained by analyzing 400 gastric cancer patients treated at three centers in Thailand and showed that elevated expression of MMP-7 is associated with clinicopathological outcomes related to poor prognosis in gastric cancer. We further observed a significant association of the variant genotype; gastric cancer patients carrying GG of MMP-7 rs11568818 had a greater increased risk of MMP-7 expression than those carrying the AA and AG genotypes. These findings suggest that MMP-7 expression can serve as a prognostic factor for unfavorable outcome in patients with gastric cancer.

Aggressive gastric cancer is characterized by invasion depth, TNM stage, and distant metastasis [12]. Most gastric cancer patients suffer from invasion of adjacent organs by gastric cancer cells. MMPs are essential enzymes that break down the ECM and subsequently allow cancer cells to migrate through degraded structures, thereby contributing to the first step in tumor development and metastasis [16]. MMP-7 is recognized as a critical player in the MMP family since it activates other MMPs (i.e., MMP-1, MMP-2, and MMP-9) for ECM degradation [17]. MMP-7 expression occurs mainly in tumor cells, and it promotes tumor progression by facilitating ECM turnover and remodeling, decreasing cell adhesion and inflammation, enhancing cell proliferation, inhibiting apoptosis, and inducing angiogenesis [18]. Elevated MMP-7 levels have been reported in multiple types of cancers, including gastric cancer [12]. The results of our study are consistent with previous reports demonstrating higher MMP-7 expression (70.75%) in gastric cancer patients. Although the prognostic value of MMP-7 in gastric cancer has been widely investigated, there are no ideal markers because the results are not consistent [12, 19]. In this study, the associations between MMP-7 expression and various clinicopathological outcomes were investigated to clarify the prognostic value of MMP-7 expression. We found that MMP-7 expression was associated with lymphatic and vascular invasion, suggesting a role for MMP-7 in invasion and metastasis in gastric cancer. MMP-7 expression also showed a statistically significant association with higher TNM staging and undifferentiated-type gastric cancer. In agreement with our study, Huang et al. demonstrated that MMP-7 plays a role in gastric cancer progression, suggesting its usefulness as a marker for identifying aggressive biological gastric cancer [20]. The findings support the hypothesis that MMP-7 expression has a crucial role in the progression and prognosis of gastric cancer.

MMP-7 also cleaves additional substrates, including osteopontin and cell-associated Fas ligand, promotes the release of TNF-*α*, and mediates E-cadherin ectodomain shedding [21]. E-cadherin is an important intercellular adhesion molecule in epithelial tissue, including gastric tissue. It plays a key role in the modulation of cell proliferation, and downregulation of E-cadherin is potentially important in facilitating the loss of differentiation and increased invasive and distant metastatic capacity of cancer cells [22, 23]. Previous studies have provided evidence that genetic mutations in undifferentiated-type adenocarcinoma lead to dysfunction of the E-cadherin gene, resulting in loss of cell adhesion and poor clinicopathological outcomes [22]. In this study, high expression of MMP-7 occurred more often in undifferentiated-type gastric cancer than in differentiated gastric cancer and showed an association with depth of invasion by the tumor and with lymph node metastasis. These results suggest that the invasiveness of gastric cancer may result from MMP-7 mediated E-cadherin cleavage.

Carcinoembryonic antigen (CEA), a glycoprotein that is involved in cell adhesion and apoptosis, is commonly used as a marker for gastric cancer [23, 24]. Previous studies have demonstrated that serum CEA levels are significantly and positively correlated with TNM stage, depth of invasion, likelihood of cure, and lymph node metastasis in gastric cancer [24]. The role of CEA in distant metastasis may be explained by CEA's acting as an adhesion molecule in invasion and metastasis, so that cancer cells that produce CEA have a greater chance of metastasis [25]. Although numerous studies have used CEA levels to predict stage, tumor progression, recurrence, and prognosis in gastric cancer [26], this marker has limited clinical utility due to its low specificity and sensitivity [27]. Therefore, several other biomarkers, including MMPs, have been suggested. Our results showed that high serum CEA levels were associated with high expression of MMP-7 protein in gastric cancer patients. Further studies comparing MMP-7 expression and CEA levels in gastric cancer are needed.

Several studies have suggested the essential importance of MMP-7 polymorphisms as a risk factor and as a marker of poor prognosis in multiple types of cancer, including gastric cancer [13, 28]. Our results show that gastric cancer patients who carry the GG genotype of MMP-7 rs11568818 were more likely to exhibit high MMP-7 expression, resulting in enhanced tumor progression and poor prognosis of these patients. Zhang et al. reported that the MMP-7-181G allele showed a higher affinity for nuclear proteins than did the -181A allele and thus enhanced the transcription of the MMP-7 gene [28]. This might explain why the 181GG genotype enhances the expression of the MMP-7 protein.

This study has some limitations. First, the study design was retrospective, and this might be a source of uncontrolled bias. Second, some patients received chemotherapy after surgery, resulting in a greater chance of survival for those patients than for those who did not receive chemotherapy; therefore, an association between MMP-7 expression and the survival time of gastric cancer patients could not be demonstrated. To obtain more conclusive results, we recommend determining the association between MMP-7 expression and the survival of gastric cancer patients in future studies. Further studies involving more patients and clinical validation are also needed.

## 5. Conclusions

The results of this study revealed high expression of MMP-7 in gastric cancer and showed a significant association of MMP-7 expression with poor clinicopathological outcome. This suggests that MMP-7 might be a potential candidate marker for identifying patients who have more aggressive gastric cancer and suggests its potential usefulness to clinicians in selecting better therapeutic approaches and conducting intensive follow-up of these patients.

## Figures and Tables

**Table 1:** Patient's demographics data among gastric cancer patients and MMP-7 status.

Patient's demographics data	MMP-7 status		*P* value
	Low expression	High expression	
Age (year ± SD)	56.03 ± 10.42	59.32 ± 12.63	0.251
Sex (male (%))	65(55.55)	125(44.16)	0.097
Underlying condition (%)			
HT	11(9.40)	22(7.77)	0.752
DM	15(12.82)	30(10.60)	0.413
Hyperlipidemia	8(6.83)	10(3.53)	0.073
Smoking	19(16.23)	40(14.13)	0.348
Alcohol	15(12.82)	20(7.06)	0.169
Family history of gastric cancer	4(3.41)	11(3.88)	0.983
MMP-7 (positive (*n*) (%))	117(29.25)	283(70.75)	0.001∗

Comparison between the groups were done by using ANOVA. ∗*p* < 0.05 considered as statistically significant.

**Table 2:** Genotype distribution of the MMP-7 gene polymorphism (-181A/G; rs11568818) related to MMP-7 expression in gastric cancer patients.

MMP-7 polymorphism (%)	MMP-7 status		OR; 95% CI	*p* value
	Low expression (*n* = 143)	High expression (*n* = 257)		
AA	105 (26.25%)	171 (42.75%)	0.87 (0.56-1.30)	0.064
AG	29 (7.25%)	38 (9.5%)	0.93 (0.82-1.47)	0.137
GG	9 (2.25%)	48 (12%)	5.87 (3.73-9.24)	0.035

Multivariate regression model analysis used to analyze the data. OR: odds ratio; CI: confidence interval. ∗Significance is set at *p* < 0.05.

**Table 3:** The association of MMP-7 status and clinicopathological outcome of gastric cancer.

Gastric mucosal pathology	MMP-7 status		OR; 95% CI	*p* value
	Low expression (*n* = 117)	High expression (*n* = 283)		
Location of tumor (%)				
Upper	16 (13.67)	44 (15.54)	0.75 (0.61-0.92)	0.229
Middle	41 (35.04)	75 (26.50)	0.71 (0.54-0.93)	0.127
Lower	60 (51.28)	164 (57.95)	0.86 (0.71-1.14)	0.573
Tumor size (%)				
<70 mm	68 (58.12)	153 (54.06)	0.67 (0.52-0.84)	0.872
≥70 mm	49 (41.88)	130 (45.93)	0.71 (0.64-0.92)	0.791
Histologic type (%)				
Differentiated	82 (70.08)	94 (33.21)	0.96 (0.67-1.08)	0.092
Undifferentiated	35 (29.91)	189 (66.78)	2.89 (1.57-4.94)	0.018∗
Lymphatic invasion (%)				
Absent	99 (84.61)	64 (22.61)	0.37 (0.14-0.76)	0.028∗
Present	18 (15.38)	219 (77.38)	9.87 (5.57-13.29)	0.019∗
Vascular invasion (%)				
Absent	89 (76.06)	52 (18.37)	0.42 (0.21-0.83)	0.017∗
Present	28 (23.93)	231 (81.62)	8.16 (4.87-10.19)	0.015∗
Pathological TNM stage (%)				
I	8 (6.83)	21 (7.42)	0.88 (0.69-0.97)	0.826
II	25 (21.36)	32 (11.30)	0.91 (0.88-1.19)	0.482
III	52 (44.44)	86 (30.38)	0.69(0.54-0.83)	0.257
IV	32(27.35)	144(50.88)	1.95 (1.04-3.08)	0.042∗
Residual tumor (%)	(*n* = 9)	(*n* = 22)		
Microscopic	3 (33.33)	8 (36.36)	0.72 (0.54-0.93)	0.628
Gross (unresectable)	6 (66.66)	14 (63.63)	0.68 (0.41-0.84)	0.712
CEA (%)				
<5.0 (ng/ml)	101 (86.32)	67 (23.67)	0.38 (0.19-0.69)	0.036∗
≥5.0 (ng/ml)	16 (13.67)	216 (76.32)	3.96 (1.68-5.27)	0.012∗

Univariate regression model analysis. ∗*p* < 0.05 considered as statistically significant.

**Table 4:** The association of MMP-7 status and clinicopathological outcome of gastric cancer.

Gastric mucosal pathology	MMP-7 status		OR; 95% CI	*p* value
	Low expression (*n* = 117)	High expression (*n* = 283)		
Lymphatic invasion (%)				
Absent	99 (84.61)	64 (22.61)	0.27 (0.16-0.72)	0.022∗
Present	18 (15.38)	219 (77.38)	8.17 (4.47-12.39)	0.017∗
Vascular invasion (%)				
Absent	89 (76.06)	52 (18.37)	0.58 (0.21-0.87)	0.036∗
Present	28 (23.93)	231 (81.62)	6.16 (4.26-9.89)	0.024∗
Histologic type (%)				
Undifferentiated	35 (29.91)	189 (66.78)	2.46 (1.31-4.52)	0.014∗
Pathological TNM stage (%)				
IV	32 (27.35)	144 (50.88)	1.48 (1.08-3.08)	0.047∗
CEA (%)				
< 5.0 (ng/ml)	101 (86.32)	67 (23.67)	0.47 (0.28-0.88)	0.041∗
≥ 5.0 (ng/ml)	16 (13.67)	216 (76.32)	5.96 (2.12-8.12)	0.026∗

Multivariate regression model analysis. ∗*p* < 0.05 considered as statistically significant.

## Data Availability

The datasets produced and analyzed in the study are contained in the paper.
